# Upward migration of a double J ureteral stent in a boy: A rare case report

**DOI:** 10.1016/j.amsu.2022.104604

**Published:** 2022-09-09

**Authors:** Maher Al-Hajjaj

**Affiliations:** Department of Urology, Aleppo University Hospital, Aleppo, Syria

## Abstract

**Introduction:**

Inserting double j ureteral stents is considered a common practice in the urology field. With the wide range of use, many complications were reported.

**Case presentation:**

We are presenting a rare case of five years old boy that had a right renal stone with moderate hydronephrosis. Inserting a double j stent was the first procedure to relieve the obstruction. Metabolic studies were negative. Twelve days later, the double j stent migrated upward. As a result, we performed an open surgery to remove the stone and the double j. During surgery, we inserted a new double j stent that was removed six weeks later. Follow-up showed no complaints.

**Discussion:**

Upward migration of a double j ureteral stent in a child is considered a rare entity. We performed open surgery to remove the ureteral stent and the stone. Such complication in a child has not been reported widely.

**Conclusion:**

We aim to report an upward migration double j ureteral stent in a boy and how we dealt with it.

## Highlights

• Double-j ureteral stents are common tools in daily urology practice.
• Many complications were reported related to inserting a double-j.
• Upward migration is one of the rare complications result in malposition of the double-j.
• Reposition or remove of the double-j as soon as possible is important to avoid more damage.

## 1 Introduction

Ureteric stenting is one of the most commonly applied urological techniques, particularly since the introduction of the ‘double J’ stent by Finney in 1972 [1].

Despite the advances and technology, the ideal stent is not available yet. A double-J stent is never without potential complications which may be minor in form of hematuria, dysuria, frequency, flank and suprapubic pain to major complications such as vesicoureteric reflux, migration, malposition, encrustation, stent fracture etc [2].

Upward migration of DJ stent is occasionally encountered in urologic practice. Ureteroscopy is usually used to remove upward DJ stent [3].

Herein, we report a rare case of upward migration of a double j ureteral stent in a boy who had a calculus disease.

This case report examines one such presentation in line with the SCARE guidelines [5].

## 2 Case presentation

**Fig. 1:**
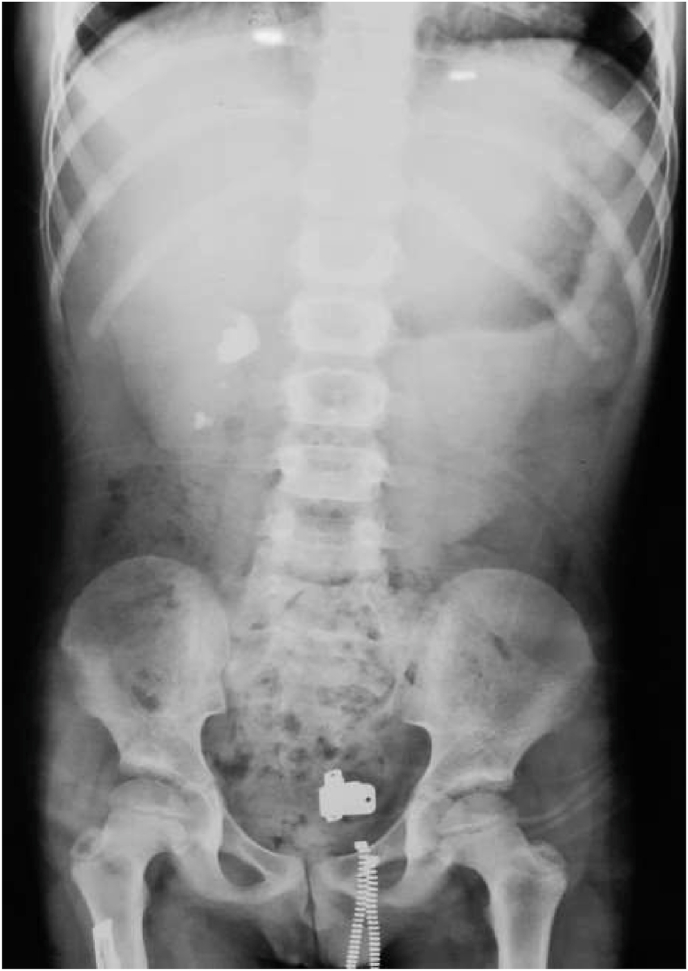
KUB image shows right renal calculus.

A 5 years old boy presented to our emergency department with 3 h of abdominal pain, nausea, vomiting, and macroscopic hematuria. Past medical history was unremarkable except for a tonsillectomy two years ago. Physical examination demonstrated abdominal tenderness mostly in the right flank. Vital signs were: blood pressure 90/40 mm/Hg, pulse 110 bpm, and temperature 37.1 °C. Abdominal and pelvis ultrasound revealed moderate right hydronephrosis with a calculus. KUB image confirmed the stone in the right kidney (Fig. 1).

Urinalysis demonstrated hematuria (+2). Urine microscopy showed the presence of red blood cells of 30–40/HPF, and white blood cells of 17/HPF. His laboratories were as follows: 9 × 105/ml white blood cell, 11 × 105gr/dl hemoglobin, 0.8 mg/dl creatinine, and 22 mg/dl urea.

**Fig. 2:**
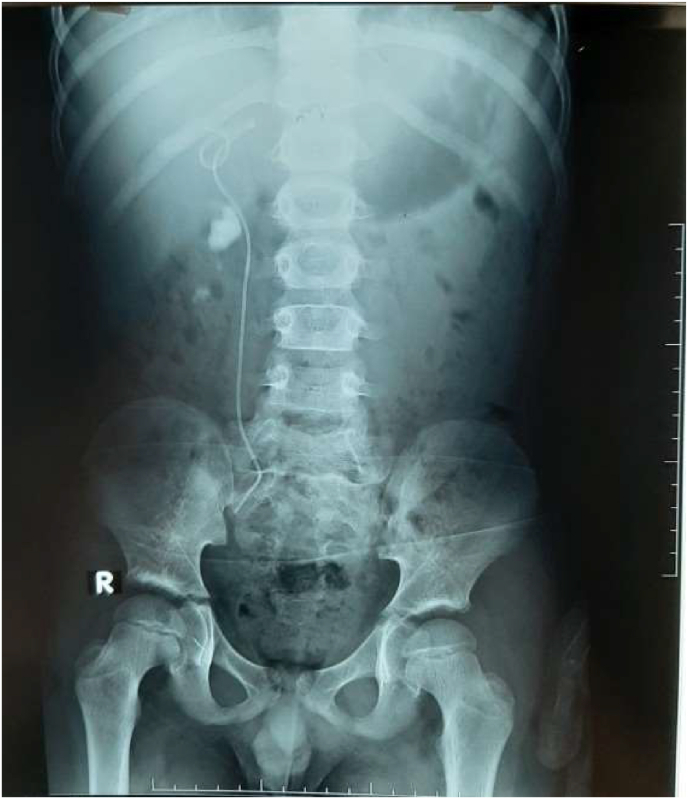
Upward migration of double j stent.

Next, we admitted the patient for fluid resuscitation and pain control. On the second day, we decided to insert the right double j ureteral stent. Under general anesthesia, we inserted a 4.7 F double j stent with no difficulties. On the same day, we sent the boy home in a good state for a scheduled stone extraction surgery after a short time. Twelve days later, he presented with right flank pain and vomiting. KUB image showed an upward migration of the double j stent (Fig. 2).

**Fig. 3:**
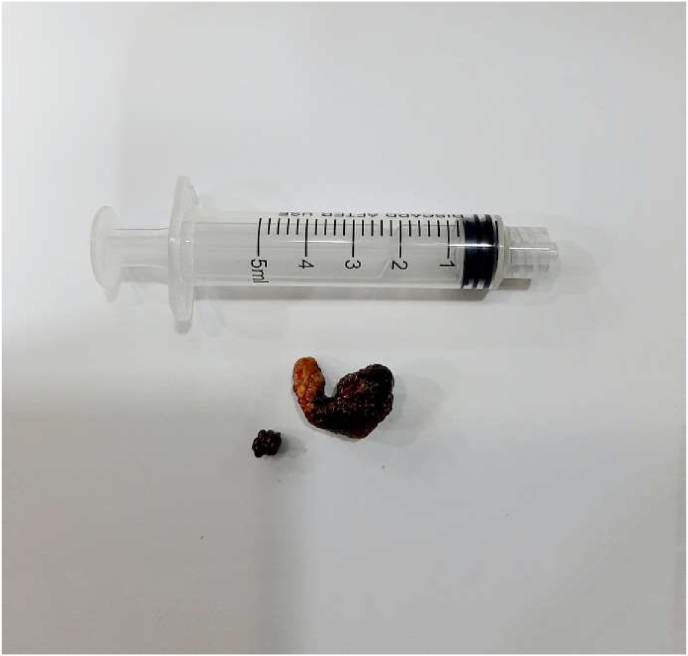
The two extracted stones from right kidney.

We decided to perform a stone surgery extraction. After taking the parents’ consent we performed open surgery. By right flank incision, we reached the right kidney. Next, we open the renal pelvis and extracted two stones (Fig. 3). Then, we inserted a 4.7 F double j ureteral stent. With 5/0 vicrel sutures, we closed the renal pelvis with a drain.

The postoperative course was uneventful. KUB image control after surgery showed a well-placed double j stent. On the third day of admission, we removed the drain. After six weeks, we removed the double j stent. Follow-up for one year revealed no recurrences (Table 1).

**Table 1:** Laboratory findings.

Wight blood cell count	Hemoglobine	Platelets	Creatinine	Urea	Glucose	Na+	K+
9 × 10^5^/ml	11 × 10^5^gr/dl	300 × 10^5^/mcl	0.8 mg/dl	22 mg/dl	85 mg/dl	142 mEq/L	4.3 mEq/L

## 3 Discussion

One of the early comlications of double J placement is a migration of the stent within the urinary tract. Gibbons et al. initially addressed the problem of downward migration of soft silicone tubing by adding barbs along the shaft of the tube, as a result there is a stent design that bears his name. Nevertheless, peristalsis may discharge a stent (especially one constructed from softer materials) from the ureter [4].

Upward migration is one of the major complications of the DJ stents. The incidence of upward migration has been reported 0.6–3.5%. The etiology of upward migration of DJ stent is multifactorial, resulting from short stent, duration of stent, the angle of distal part of stent <180°, placement of stent in upper pole instead of pelvis [3].

A similar phenomenon and mechanism of migration is seen in patients with a pacemaker—Twiddler's syndrome. This is a rare complication arising from mechanical manipulation of the pacemaker device by the patient, leading to displacement of the pacing leads from the ventricles [6].

In our case, we had five years old boy who had a right renal stone causing right flank pain and hydronephrosis. Stent placement was the first procedure to reduce hydronephrosis and relieve the pain. Unfortunately, we had a stent migration after 12 days of insertion.

In that scenario, we decided to perform the definitive surgery. The boy underwent right renal calculus and double j stent extraction surgery. Next, we inserted a new double j stent which was removed later with no complications.

## 4 Conclusion

With the wide range of indications of inserting double j ureteral stents, it has many complications. Good instructions make decisions, and scheduled follow-ups will reduce the rate of these complications.

## Ethical approval

N/A.

## Sources of funding

The article has no funding source.

## Author contribution

Maher Al-Hajjaj: is the only author.

## Registration of research studies

N/A.

## Guarantor

Maher Al-Hajjaj.

## Consent

Written informed consent was obtained from the patient's parents for publication of this case report and accompanying images. A copy of the written consent is available for review by the Editor-in-Chief of this journal on request.

## Declaration of competing interest

N/A.
